# Correction of symbrachydactyly: a systematic review of surgical options

**DOI:** 10.1186/s13643-023-02362-7

**Published:** 2023-11-16

**Authors:** A. Bartsch, D. Nikkhah, R. Miller, K. Mende, S. E. R. Hovius, A. Kaempfen

**Affiliations:** 1 Paediatric Orthopaedic Surgery, University Children’s Hospital Basel, Spitalstr. 33, CH-4056 Basel, Switzerland; 2 Orthopaedic Surgery and Traumatology, University Hospital Basel, Spitalstr. 21, CH-4031 Basel, Switzerland; 3 Department of Plastic and Reconstructive Surgery, Royal Free NHS Foundation Trust, London, UK; 4 Department of Plastic and Reconstructive Surgery, St George’s Hospital, Blackshaw Road, London, SW17 0QT5 UK; 5 Plastic, Reconstructive, Aesthetic and Hand Surgery, University Hospital Basel, Spitalstr. 21, CH-4031 Basel, Switzerland; 6 Department of Plastic, Reconstructive and Hand Surgery, Radboud University Medical Centre, Geert Grooteplein Zuid 10, 6525 GA Nijmegen, The Netherlands

**Keywords:** Symbrachydactyly, Phalangeal transfer, Toe transfer, Distraction osteogenesis, Syndactyly, Congenital

## Abstract

Symbrachydactyly is a rare congenital malformation of the hand characterized by short or even absent fingers with or without syndactyly, mostly unilaterally present. The hand condition can vary from a small hand to only nubbins on the distal forearm. This study aims to systematically review the surgical management options for symbrachydactyly and compare functional and aesthetic outcomes.

The review was performed according to the PRISMA guidelines. Literature was systematically assessed searching the Cochrane Library, PubMed, Embase, and PROSPERO databases up to January 1, 2023. Studies were identified using synonyms for ‘symbrachydactyly’ and ‘treatment’. Inclusion criteria were the report of outcomes after surgical treatment of symbrachydactyly in humans. Studies were excluded if they were written in another language than English, German, or French. Case reports, letters to the editor, studies on animals, cadaveric, in vitro studies, biomechanical reports, surgical technique description, and papers discussing traumatic or oncologic cases were excluded.

Twenty-four studies published were included with 539 patients (1037 digit corrections). Only one study included and compared two surgical techniques. The quality of the included studies was assessed using the Modified Coleman Methodology Score and ranged from 25 to 47. The range of motion was the main reported outcome and demonstrated modest results in all surgical techniques. The report on aesthetics of the hand was limited in non-vascularized transfers to 2/8 studies and in vascularized transfers to 5/8 studies, both reporting satisfactory results. On average, there was a foot donor site complication rate of 22% in non-vascularized transfers, compared to 2% in vascularized transfers. The hand-related complication rate of 54% was much higher in the vascularized group than in the non-vascularized transfer with 16%.

No uniform strategy to surgically improve symbrachydactyly exists. All discussed techniques show limited functional improvement with considerable complication rates, with the vascularized transfer showing relative high hand-related complications and the non-vascularized transfer showing relative high foot-related complications.

There were no high-quality studies, and due to a lack of comparing studies, the data could only be analysed qualitatively. Systematic assessment of studies showed insufficient evidence to determine superiority of any procedure to treat symbrachydactyly due to inadequate study designs and comparative studies. This systematic review was registered at the National Institute for Health Research PROSPERO International Prospective Register of Systematic Reviews number: CRD42020153590 and received no specific grant from any funding agency in the public, commercial, or not-for-profit sectors.

**Level of evidence**

I.

**Systematic review registration**

PROSPERO CRD42020153590.

**Supplementary Information:**

The online version contains supplementary material available at 10.1186/s13643-023-02362-7.

## Introduction

Symbrachydactyly is a rare non-inherited congenital upper limb anomaly (CULA), affecting boys and girls equally, with an incidence of 1.19/10,000 live births [79]. It is characterized by longitudinal growth disturbance and webbing of the fingers, which is mostly unilateral [27], either or not associated with malformations of muscles around the shoulder and the thoracic cage [1]. There is a large spectrum of severity depending on the evolution of different classification systems describing varying phenotypes of symbrachydactyly [2, 18, 41, 50, 61, 74, 76]. The term symbrachydactyly has been used to describe a malformation that overlaps with transverse and central deficiency, brachymetacarpia, brachyphalangism, and oligodactyly [43]. Different classifications have been made to define the varying degrees [2, 18, 41, 50, 61, 74, 76]. The oldest classifications by Pol [66], later modified by Blauth and Gekeler [2], were mainly based on morphological characteristics. A hypoplastic hand, brachymesophalangy, assimilation hypophalangy, and syndactyly was termed in 1974 by Letsune as “typical” symbrachydactyly. The Oberg-Manske-Tonkin (OMT) classification [25] defines symbrachydactyly as abnormal axis formation, i.e. in the proximo-distal axis in the spectrum with ectodermal elements (I-A-1-ii-b for the limb and I-B-1-ii for the handplate), and the close phenotype transverse arrest is classified also in the proximal–distal axis but without ectodermal elements (I-A-1-iii-b for the limb and I-B-1-iii for the handplate). In contrast, in 2015, the Japanese Society for Surgery of the Hand considered symbrachydactyly a transverse formation failure [32].

Symbrachydactyly is associated with functional and cosmetic impairment. Characteristic components of the disease include syndactyly, brachydactyly, unstable digits, and a lack of digits or parts of digits with often impaired pinch/opposition [27]. There are surgical and nonsurgical management options to improve function [17, 20, 21, 24, 54, 58], with increasing degrees for surgical complexity. Digital reconstruction can be performed by enlarging the present digits or by bringing new tissue to the shortened digit [17, 20, 54]. The following options are the most used reconstruction options to address both functional and aesthetic aspects:

1. *Free non-vascularized toe phalanx transfer (FPT)*, which involves removing a periosteum-covered proximal phalanx with growth plate from a toe and transferring it non-vascularized to the empty finger skin pocket [24]
2. *Free vascularized toe to hand transfer (FTT)*, which involves the removal of toes in total, with or without metatarso-phalangeal joint for finger reconstruction [17, 20, 48].
3. *Distraction lengthening*, in which present shortened bones are lengthened with an external fixator [42, 54, 62, 65]. This procedure is sometimes combined with free non-vascularized toe phalanx transfer, which is then called distraction augmentation manoplasty [58, 64].
4. *Syndactyly release*, in which skin and soft tissue connections are released, to enhance functional and aesthetic appearance by deepening web spaces. This is often combined with FPT or FTT [21, 28].

Optimal treatment of congenital aphalangism or severely hypoplastic digits is subject to controversial debate, and to date, there is no evidence-based management of symbrachydactyly treatment available. There is much debate on how symbrachydactyly patients benefit from vascularized or non-vascularized procedures and when one procedure should be selected over another. The gain through surgery is not always certain, and procedures are associated with risks such as instability, stiffness, skin necrosis, and donor site morbidity [58].

This study aims to systematically review the surgical management options for symbrachydactyly and compare functional and aesthetic outcomes, with a focus on the comparison between free non-vascularized toe phalanx transfer and free vascularized phalangeal transfers.

## Material and methods

The review was registered on PROSPERO (International Prospective Register of Systematic Reviews, number: CRD42020153590). We made several amendments to the systematic review protocol. We have added the French language to our inclusion criteria as a substantial number of articles was in this language. Due to the limited quantity and heterogeneity of available studies, we did not limit studies to one specific outcome but included all studies reporting on functional, aesthetic, lengthening, or complication-related outcomes. We included distraction lengthening and syndactyly release procedures to report on all relevant treatment methods. The Modified Coleman Methodology Score [10, 71] was used instead of the ROBINS-I to assess the risk of bias, as this assessment method reports on the quality of reported outcomes and rehabilitation, which we valued both important for this clinical treatment outcome review.

The literature search was performed according to the PRISMA guidelines [46], searching the Cochrane Library, PubMed, Embase, and PROSPERO databases until January 1, 2023, without a limit to the year of publication.

‘Free-text term’ using synonyms for ‘symbrachydactyly’ and ‘treatment’ was used (Supplement 1). No filters, limits, or restrictions were additionally used. Bibliographies of included studies were reviewed for relevant additional studies not identified in the primary search. Authors were not contacted. Search results for databases were merged and deduplicated with the help of Covidence (https://get.covidence.org/).

Studies were included if they reported outcomes of surgical treatment of symbrachydactyly. Authors had to name the diagnosis symbrachydactyly. Synonymous or similar definition diagnosis terms were not included. Prospective and retrospective, descriptive, and analytic studies on humans were eligible for inclusion. Included study types were randomized or quasi-randomized trials and observational study designs, including systematic reviews and meta-analyses of these study types. Studies were included if they reported one or more of the following outcomes: functional outcomes (objective or subjective), cosmetic outcomes, overall outcome scores, and patient satisfaction. Inclusion was limited to the English, German, or the French language, and only studies in humans were selected. Only scientific articles were screened.

Case reports, letters to the editor, studies on animals, cadaveric, in vitro studies, biomechanical reports, surgical technique descriptions, and papers discussing traumatic or oncologic cases were excluded. Books, websites, or videos were excluded.

Two of the authors (A. B. and A. K.) independently screened titles and abstracts of identified studies and discarded studies unrelated to the research objective. Full texts of the relevant papers were examined to further assess eligibility for data extraction. Authors compared and discussed the final list. Any disparities regarding inclusion of articles between authors were thoroughly discussed in order to reach a joint decision.

Data were collected on Excel sheets by A. B. and A. K. independently. The combined final integrated sheet of the findings can be found in the appendix (Supplement Tables 1, 2, 3 and  4). Data was extracted from the studies according to the variables in the supplement. Complication rates were defined as the primary outcome. Secondary outcomes were reports on functional, aesthetic, and lengthening results. Only qualitative analysis was performed due to the limit of available studies. Studies were grouped according to treatment method and presented outcome. Comparison was made by available data only and due to inconsistent reporting methods only descriptive. We were only able to check for plausibility of the studies by provided numbers, which were scarce.

The quality of the included studies was independently assessed by two authors (A. B. and A. K.) using the Modified Coleman Methodology Score [10, 71]. The total score reaching from 0 to 100 is based on 10 subsections, allowing a reproducible and relevant systematic review of outcomes. A score of 100 indicates that the study largely avoids chance, various biases, and confounding factors. The same two authors rated the outcome-level certainty using the Grading of Recommendations Assessment, Development and Evaluation (GRADE) approach [72].

## Results

The initial search generated in total 454 studies. After the removal of duplicates, two reviewers independently screened titles and abstracts of 449 studies, of which 396 studies were excluded due to predefined inclusion and exclusion criteria. Of the remaining 53 full texts reviewed, 29 studies were excluded; of which 18 were a review or surgical technique description without information on outcome [3, 5, 7, 12, 14, 15, 26, 27, 35, 37, 53, 57, 58, 63, 64, 79], six were case reports [9, 34, 36, 39, 56, 59], two studies were written in another language than English, German, or French [11, 16], two studies reported on findings from the same study population [4, 19], and one study was excluded as it involved animals [38] (Fig. 1).

**Fig. 1:**
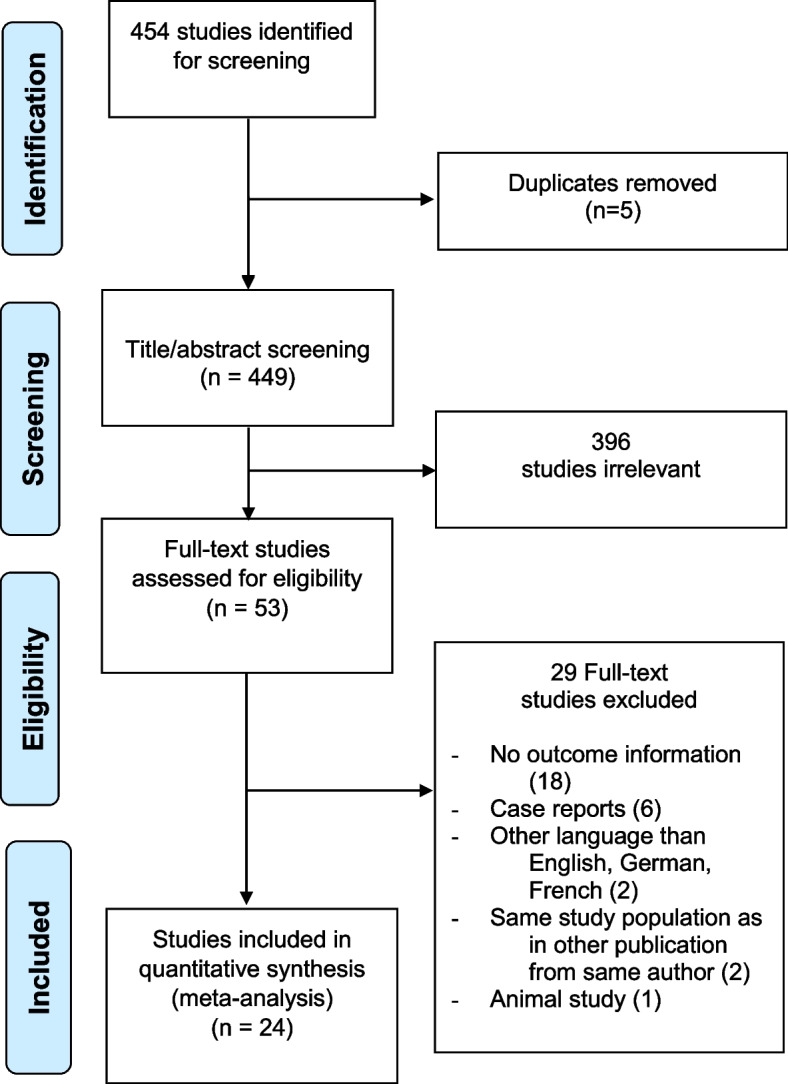
PRISMA diagram

Twenty-four studies published between 1988 and 2022 were included in our systematic review (Table 1), including patients from Germany [6, 13, 23, 30, 31, 49, 77] (29%), Japan [33, 40, 52, 54] (17%), the UK [8, 22, 60] (13%), France [17, 17, 20, 20, 44, 78] (17%), China [45, 73] (8%), the USA [48] (4%), Australia [68] (4%), Sweden [70] (4%), and India [69] (4%). All studies were retrospective case series (*n* = 15) or retrospective cohort studies (*n* = 9). No prospective comparing studies meeting our inclusion criteria were found. The Modified Coleman Methodology Score of these studies ranged from 25 to 47 (additional file 1). We rated the certainty of evidence as very low for all the reported outcome complications and functional, aesthetic, and lengthening results (Supplement 3).

**Table 1:** Characteristics of included studies

Author (year)	Treatment method	Included patient diagnosis	Number of patients (digit elongations)	Age in months (mean)	Female (%)	Average follow-up time (months)
Buck-Gramcko (1990) [4]	**Non-vascularized** transfer	Symbrachydactyly and constriction ring syndrome. Finger devoid of a bony skeleton at the level of the proximal phalanges or intermediate large bone defects in digits (mainly thumb)	40 (69)	Range 7 months to 17 years	Not reported	36
Cavallo (2003) [8]	**Non-vascularized** transfer	Symbrachydactyly, constriction band syndrome, aphalangia	22 (64)	15	41	59
Deutinger (1989) [13]	Web reconstruction	Symbrachydactyly and syndactyly	29 (62)	144	34	-
Foucher (2001)-V [17]	**Vascularized** transfer	Symbrachydactyly (*n* = 45), transverse deficiency (*n* = 2), thumb hypoplasia (*n* = 5), miscellaneous (*n* = 6)	58 (65)	16	Not reported	62
Foucher (2001)-D [20]	Distraction osteogenesis	Symbrachydactyly (*n* = 21), clinodactyly (*n* = 5), Apert (*n* = 4), brachydactyly (*n* = 7), others (*n* = 4)	41 (41)	109	Not reported	8
Garagnani (2012) [22]	**Non-vascularized** transfer	Symbrachydactyly (*n* = 33), constriction ring syndrome (*n* = 3), thumb hypoplasia (*n* = 3), perinatal subclavian venous thrombosis (*n* = 1)	40 (126)	32	58	122
Gohla (2005) [23]	**Non-vascularized** transfer	Symbrachydactyly	48 (113)	43	44	72
Hierner (1998) [30]	Distraction osteogenesis	Symbrachydactyly (*n* = 2), adactyly with transversal defect and acrosyndactyly (*n* = 3)	5 (9)	14.6	Not reported	12
Iba (2012) [33]	Web reconstruction	Symbrachydactyly (*n* = 2), cleft hand (*n* = 1), constriction band syndrome (*n* = 1)	2 (3)		20	49.5
Hulsemann (2002) [31]	**Vascularized** transfer	Symbrachydactyly of the peromelic type	11 (22)	48	Not reported	64
Kawabata (2018) [40]	**Non-vascularized** transfer	Symbrachydactyly	29 (54)	18	Not reported	89
Leca (2008) [44]	**Non-vascularized and vascularized** transfer	Symbrachydactyly	3 (7)	14	0	84
Li (2013) [45]	Web reconstruction	Symbrachydactyly of the short finger type	34 (120)	31	38	12
Lister [48]	**Vascularized** transfer	Congenitally deficient thumbs: Symbrachydactyly (*n* = 3), constriction ring syndrome (*n* = 3), transverse arrest (*n* = 6)	12 (12)	36	Not reported	48
Mann (2016) [49]	Distraction osteogenesis	Symbrachydactyly (*n* = 32) and amniotic band syndrome (*n* = 10)	60 (71)	104	Not reported	-
Matsuno (2004) [52]	Distraction osteogenesis	Symbrachydactyly (*n* = 3) amniotic band syndrome, hypoplastic thumb, hypoplasia of the small finger, cleft hand, metacarpal synostosis, and brachymetacarpia	15 (23)	Not reported	Not reported	59
Miyawaki (2002) [54]	Distraction osteogenesis	Symbrachydactyly (Müller type D)	4 (7)	88	25	-
Nikkhah (2016) [60]	**Vascularized** transfer	Symbrachydactyly (*n* = 12), trauma (*n* = 3)	12 (19)	28	Not reported	78
Richardson (2004) [68]	**Vascularized** toe to hand transfer	Symbrachydactyly (*n* = 7 monodactylic form, *n* = 6 adactylic)	13 (18)	31	Not reported	-
Sabapathy (2021) [63]	**Non-vascularized** transfer	Symbrachydactyly (*n* = 18), bilateral transverse deficiency (*n* = 1)	19 (40)	Not reported	37	53
Schenker (2007) [70]	**Vascularized** transfer	Symbrachydactyly (*n* = 8)	8	34.5	Not reported	57
Shen (2022) [73]	Web reconstruction **non-vascularized** transfer	Symbrachydactyly (*n* = 16), symbrachydactyly (congenital short finger (*n* = 4), oligodactylic type (*n* = 6), monodactylic type (*n* = 6), peromelic type (*n* = 3)), and ring constriction syndrome (*n* = 1)	16 (72) 20 (56)	Median 24.4 months (range 7 to 84)	44	19
Unglaub (2006) [77]	**Non-vascularized** transfer **Vascularized** transfer	Symbrachydactyly (congenital short finger (*n* = 4), oligodactylic type (*n* = 6), monodactylic type (*n* = 6), peromelic type (*n* = 3)), and ring constriction syndrome (*n* = 1). Symbrachydactyly (*n* = 2), constriction ring syndrome (*n* = 3), transverse absence (*n* = 9)	20 (56) 14 (28)	58	Not reported	42
Van Holder (1999) [78]	**Vascularized** transfer	Symbrachydactyly (*n* = 2), constriction ring syndrome (*n* = 3), transverse absence (*n* = 9)	14 (28)	44	29	-

A total of 555 patients comprising 1109 digital corrections were included. Patients follow-up period was 50 years in total and ranged from 1969 to 2020. The mean age of patients was 46 months at the time of surgery, and 36% were female (Table 1). Nine studies [23, 30, 31, 40, 45, 54, 68, 70, 73] described outcomes on symbrachydactyly only. In the remaining studies, symbrachydactyly cases were included alongside other congenital upper limb anomaly conditions such as constriction band syndrome [6, 8, 22, 48, 49, 52, 77, 78] (*n* = 8), aphalangia, syndactyly, thumb hypoplasia, and transverse arrest. Some studies described outcomes on different congenital diseases; however, the outcomes for symbrachydactyly were analysed separately (Table 1). Eight studies in total described non-vascularized toe-to-hand transfers [6, 8, 22, 23, 40, 44, 69, 77], 8 studies vascularized toe-to-hand transfers [17, 20, 31, 44, 48, 60, 68, 70, 78], five looked at distraction osteogenesis [17, 20, 30, 49, 52, 54], and four examined web release [13, 33, 45, 73]. Only one study compared non-vascularized phalangeal transfer and vascular toe transfer on a single patient [44]. No other study compared different surgical treatments. Follow-up time ranged from 12 to 122 months postoperatively, with a median follow-up time of 4.9 years. Short-term follow-up (≤ 3 years) was reported in 5 studies [6, 17, 20, 30, 45, 73], middle-term follow-up (3–5 years) in 7 studies [8, 33, 48, 52, 69, 70, 77], and long-term follow up (> 5 years) in 7 studies [17, 20, 22, 23, 31, 40, 44, 60]. Five authors did not provide information on follow-up time [13, 49, 54, 68, 78]. Reports on outcome were in all studies made by the treating clinical group, and no independent researchers were involved. Moreover, information on post-operative treatment was very limited, and a precise description of postoperative rehabilitation was provided by one study only [31].

## Functional outcomes of the hand

For non-vascularized transfers, the range of motion (ROM) was the main reported outcome (Supplement Table 1). This analysis was performed by the authors and demonstrated modest results (average 10° to average 60°) [23]. An improvement of functional performance was confirmed in the majority of patients, when asked about overall satisfaction with the postoperative function [23, 40, 69, 77]. Buck-Gramko and colleagues carried out an age-specific sub-analysis of the ROM in the new joint, with better results in the younger group (average ROM with age ≤ 18 months 35°; 19–48 months 10°; > 48 months 15°) [6]. For vascularized transfers, more neurovascular functions were investigated including sensation [17, 20, 31, 78], pincer strength [31], and sweating [48]. The overall results were simply reported as satisfactory and sweating [48]. The overall results were simply reported as satisfactory without a specific measurement table, and up to 77% of the parents were happy with the function of the hand [68]. Distraction osteogenesis showed limitations in improving thickness of the digit or joint motion [17, 20] but was able to successfully improve pinch power [54] (range, 0.4 to 2.2 kg). A pinch grip was not achievable in all patients [30].

Three studies reported on web reconstruction only (Supplement Table 3). Deutinger described good results in improving the range of motion without further detail [13]. Li reported a 94% parents’ satisfaction rate with the postoperative function of the hand [45]. Shen combined web reconstruction with rotation osteotomy and reported that all reconstructed thumbs had functional opposition and were used by patients in daily activities [73, 73].

## Aesthetic outcomes of the hand

The report on aesthetics of the hand in non-vascularized transfers was limited to two of eight studies [69, 77], as most of the studies focused on the donor feet (Supplement Table 1). Sabapathy et al. were the only study to use a validated outcome questionnaire (Michigan Hand Questionnaire). Children gave higher scores (78.1/100; 0–100 from worse to normal) than parents (63.3/100) [69]. Unglaub reported only a 50% improvement in patients’ reported self-confidence of the child [77].

Only two of the five studies investigating aesthetics of vascularized transfers reported on aesthetic results of the hand, whereas all of them reported on the aesthetic results of the foot (Supplement Table 2). Richardson and Van Holder reported high satisfactory levels with the appearance of the hand [68, 78].

In distraction lengthening, no study reported on subjective aesthetic outcome. One study described results for aesthetic appearance as reported by the surgeon, which was undesirable [52].

Li described that in web reconstruction, 76% (n/N) of parents were satisfied with the cosmetic appearance, being the only reference for web reconstruction in symbrachydactyly [45] (Table 1).

### Lengthening results

In non-vascular phalangeal transfer, digital growth was documented as radiographic closure of the growth plate or millimetre growth (Supplement Table 1). Studies agreed that younger age is related to higher average growth, and growth rates are highest in an age under 18 months [6, 8, 23]. One study reported finger length compared to the contralateral side, reporting a finger length of 71.8% compared to the contralateral proximal phalanx of the foot [69].

In vascularized transfers, growth was described as similar to the contralateral toe side, with premature growth plate closure in only 4/72 children [17, 20, 78] (Supplement Table 2).

In distraction osteogenesis, the lengthening results reported were 20.4 mm [30], 18 mm [49], 22 mm [54], or 48% of the contralateral side, without specification to which finger [52].

### Donor site (foot) results and complications

On average, there was a 22%-foot donor site complication rate in non-vascularized transfers, compared to a 2% foot-related complication rate in vascularized transfers (Table 2).

**Table 2:** Comparison *donor site* (foot)-related complications in vascularized vs. non-vascularized transfers

Vascularized transfer	Non-vascularized transfer
• 0% complications: no morbidity in the donor feet was noted. All patients were able to run, and no neuromas were noted [17, 20] • 9% complications: one child had little problems after walking several hours on asphalt [31] • 0% complications: no difficulty was encountered at the donor feet [48] • 0% complications: 100% parents happy with function and appearance of foot donor site [68] • 0% complications: no foot problems were reported [78] • 8% complications: minor wound dehiscence (*n* = 1) [60] **2% foot-related complications**	• 13% complications: 9 relevant toe shortenings in need of reoperation [6] • 8% complications: unacceptable deformity of > 8 mm (*n* = 3), accidental transection flexor tendon (*n* = 2) [8] • 100% complications: 100% toe instability [22] • 6% complications: hypertrophic scarring (*n* = 1), instability (*n* = 1), axis deviation (*n* = 1) [23] • 0% complications: no complications reported [69] • 5% complications: hypertrophic scar (*n* = 1) [77] **Average 22% foot-related complications**

In non-vascularized transfers, toe shortening was reported in all studies, describing outcomes of the feet. Functional impairment and aesthetic issues were described in up to 100% of the patients [23] and up to 93% patient dissatisfaction [22]. The main donor site complications are toe shortening [6, 22, 69], instability of the toe remainders [23], and axis deviation [23]. Cavallo et al. showed that the middle phalanx of the toe seems to be more robust than the proximal phalanx in terms of resorption. Garagnani reported emotional disorders to foot appearance [22], and Hulsen described that no child had cosmetic issues concerning the donor site [31]. Buck-Gramcko explained that surgery-related severe shortening (> 8–12 mm) only was seen when flexor–extensor interposition had not been performed. No functional gait disturbance was noted.

In vascularized transfers, only one study described an aesthetic issue, a hypertrophic scar of the foot [78]. Only one child had some difficulty walking on asphalt for several hours [31]. All others stated very good results at the donor feet with no morbidity or cosmetic issues, and no reported complications occurred. Richardson outlined that 100% of the parents of 13 patients in his study were happy with the appearance of the donor site foot [68].

### Complications hand

A 16% hand-related complication rate was reported for non-vascularized phalangeal transfers across all studies (Table 3). The most commonly reported complication was bone resorption of the transplanted phalanx, reported in 33/170 patients [6, 22, 23, 44, 69, 77]. Bone resorption of the transplanted phalanx especially occurred in trimmed or partially explanted phalanges [23]. Digital complications were at the highest when the skin and soft tissue envelopes were scarred and limited [8]. In one case, tight closure even led to toe loss after wound necrosis with subsequent infection [8]. Similarly, Gohla reported that in two cases with skin necrosis and subsequent infection, the transplanted phalanges had to be removed [23], and Unglaub et al. reported that one wound infection led to the loss of the phalanx [77]. Four of 585 non-vascularized transplanted phalanges (0.7%) were therefore lost due to skin necrosis and infection. Both studies also included constriction band syndrome, and we could not differentiate if these cases with tight soft tissues were symbrachydactyly or possibly the constriction band cases. Other less reported complications included wound issues (8%) [6, 8, 23, 40, 69, 77], dislocation (1%) [6, 8], and infection (1%) [23, 77].

**Table 3:** Comparison *recipient site* (hand)-related complications in vascularized vs. non-vascularized transfers

Vascularized transfer	Non-vascularized transfer
• 6% complications: skin necrosis (*n* = 2; 1 partial, 1 full), instability (*n* = 2) [17, 20] • 100% complications: skin necrosis (*n* = 3; 1 partial, 2 full) with bad sensibility. All 22 patients were tenolysed 5 to 24 months postoperatively. Second tenolyse required (*n* = 2), correction osteotomy (*n* = 2), tendon transposition (*n* = 2), CMCJ arthrodesis of the radial toe (*n* = 1) [31] • 8% complications: tenolysis required (*n* = 1) [48] • 56% complications: wound breakdown (*n* = 1), skin graft loss (*n* = 1), K-wire infection (*n* = 1), in the long term, tenolysis was required (*n* = 6), development of a hammer toe (*n* = 1) [68] • 100% complications: secondary operations were required in 100% (14 patients). These included tenolysis, repair of tendon rupture, secondary tendon grafting or transfer, opponensplasty, web space deepening, metacarpal osteotomy, and ligamentplasty for joint instability [78] • 42% complications: tenolysis (*n* = 5), wound infection (*n* = 3) [60] **Average of 54% hand-related complications**	• 43% complications: limited postoperative growth, so secondary lengthening was performed (32%), wound issues (*n* = 5), subluxation (*n* = 2), resorption (*n* = 1) [6] • 3% complications: wound necrosis (*n* = 1), infection (*n* = 1) [8] • 6% complications: resorption (*n* = 7) [22] • 25% complications: resorption (*n* = 22), skin necrosis (*n* = 4), infection (*n* = 2) [23] • 9% complications: partial necrosis (*n* = 5) [40] • 5% complications: skin necrosis (*n* = 1), resorption (*n* = 1) [69] • 5% complications: wound issues with partial skin necrosis (*n* = 2), wound infection (*n* = 1) with total resorption [77] • 33% complications: phalanx resorption (*n* = 1) [44] **Average of 16% hand-related complications**

Overall, the hand-related complication rate of 54% was much higher in the vascularized group than in the non-vascularized transfer. Van Holder and colleagues reported a 100% complication rate for vascularized toe transfer [78], including tenolysis, tendon rupture, secondary tendon grafting or transfer, opponensplasty, webspace deepening, metacarpal osteotomy, and ligamentoplasty for joint instability. The authors did not describe the frequency of these complications. The remaining studies [17, 20, 31, 48, 68] reported vascular problems that required reoperation as skin necrosis [17, 20, 31, 68] or required tenolysis [31, 48, 68]. Only two studies reported complete toe loss due to vascular complications; Foucher et al. described in one child with bilateral monodactylous hands and bilateral tibial aplasia toe loss due to failed revascularization [18]. Hülsemann et al. described in two cases an arterial spasms, which lead to toe loss [31]. Therefore, 2/200 (1%) of the transferred toes could not be salvaged, and transferred toe loss occurred. In addition, 13% required a secondary tenolysis [31, 48, 68], and 4% axis malformation was noted, 2% with instability, and in 1% infections.

Of the five studies performing distraction osteogenesis, complications included infection (5/113–4%), early consolidation (11/113–10%), late consolidation (2/113–2%), 4/113 (4%) axis deviation, 4/113 (4%) re-fracture, 3/113 (3%) excessive pain, and 1/113 (1%) joint dislocation, and 1/113 (1%) tendon dislocation. The average complication rate was therefore 38% (Supplement Table 4).

For web reconstruction, reported complication rates were high, with 18% recurrence of syndactyly [13] and partial skin necrosis [45]. Syndactyly recurrence occurred in 9 divided pairs of fingers; in 7 cases, a split thickness skin graft was used. The use of split thickness skin grafts resulted in a 60% recurrence rate, whereas the use of full-thickness skin graft merely led to 7.5% recurrence rate (Supplement Table 4) [13].

### Surgical timing

Buck-Gramcko [6], Cavallo [8], and Gohla [23] divided their patients treated with non-vascularized toe transfers into three groups according to age at surgery. Patients receiving transfers between 18 and 48 months according to the authors reported the best functional outcomes without detailing measurements. Surgery at a younger age results in less bone resorption [8], and the transplanted toe phalanx physis is more likely to remain open in younger patients [24, 67]. Yet, all ages show disappointing phalangeal growth after transfer [8, 36, 75].

## Discussion

Over the years, a large number of digital reconstructions for symbrachydactyly were reported and summarized in this systematic review.

However, all included studies had a retrospective observational design and reported on various outcome measurements without control groups. Moreover, they did encompass a heterogeneous patient population. Therefore, this review only shows limited evidence on treatment modalities for symbrachydactyly.

For cases of severe and functionally limiting symbrachydactyly without pinch grip, free vascular or non-vascular toe-to-hand transfers are accepted treatment options despite the substantial complications found in this review [68, 69]. In cases of circumscribed deficits, distraction osteogenesis or web reconstruction may be advantageous.

For functional outcomes, the studies mainly demonstrated modest results in all surgical techniques. The aesthetics of the hand reported satisfactory results. On average, there was a foot donor site complication rate of 22% in non-vascularized transfers, compared to 2% in vascularized transfers. The hand-related complication rate of 54% was much higher in the vascularized group than in the non-vascularized transfer with 16%.

From the 23 studies identified, only one study compared retrospectively outcomes of vascularized and non-vascularized surgery directly, respectively, and used both techniques on the same patients with complementary indications [44]. All included studies show limited functional improvement and specific complication rates.

Surgical reconstruction is frequently performed before children with symbrachydactyly are old enough for validated functional tests, and objective assessment of infants is difficult. In our review, most authors described the postoperative range of motion as functional results [6, 8, 13, 17, 20, 52, 69, 78], yet no preoperative measurements were mentioned. The lack of preoperative data renders the evaluation of functional improvement after the procedure impossible. Most children are at an age where cooperation during examination is very limited and active functional testing is challenging. Observation during game playing and the ability of the patient to handle objects may be a better approach and more significant than range of motion measurements to justify functional enhancement surgery. Comparability of outcomes though is largely compromised.

This raises the challenge of subjective outcome measures. The patient-reported outcome measures (PROMs) used to assess patients with congenital hand differences postoperatively in the included studies were not validated for children. They were mostly limited to the general question of overall satisfaction with postoperative function, cosmetic appearance, or justifiability of the surgery. A validated PROM on children to embrace the biosocial model of illness would be beneficial to improve these dimensions in future work. The International Consortium of Health Outcomes Measurement (ICHOM) can give support in globalizing and helping standardizing subjective patient outcome evaluation in children with rare diseases as congenital malformation of the hand with their standard set of minimal required outcome measures for comparability for future studies.

Nevertheless, given the reported high level of functionality of children’s hands described in daily life and digit opposability and stability, we assume that hand function was improved regardless of treatment in most patients.

The cosmetic aspects of paediatric hand reconstruction should be acknowledged to improve the children’s social well-being. Only minimal data in the studies reviewed are available and focused mostly on the foot. Poor aesthetic outcomes can cause social withdrawal and reduce participation in daily life [29]. Further studies on aesthetic outcomes and psychological effects would be desirable. Balancing functional versus cosmetic outcomes is challenging, and the surgical goal should be chosen carefully and decided individually.

Overall, severe donor site complications were infrequent. Although studies of countries were included where flip-flops are the shoes of choice and donor sites are visible, it did not affect functional results with gait disturbance or toe instability [22, 23, 77]. No overall functional gait impairment was reported, which is the ultimate outcome for most patients. It should be highlighted that donor site complications were not documented or assessed in some of the included studies reporting on vascularized and non-vascularized procedures, which increases the risk of bias in outcome reporting. Differences in donor site closure might change the outcome, but studies comparing these are lacking, and due to different donor site measurements, this cannot be assessed sufficiently in a meta-analysis. Regardless, the high foot morbidity rate of 22% in non-vascularized transfers vs. 2% in vascularized transfers should be considered in the decision-making process for the operation of a child.

The age of included patients ranged from a few months to several years at the time of surgery. In the included studies, the authors performed transfers around 4 years of age, but Lister has described toe transfers as early as 6 months to 1 year of age [47]. Optimal timing for vascularized toe-to-hand transfers remains subject to controversial discussions. Advantages and disadvantages of a young patient’s age must be weighed against each other and are dependent on patients’ and surgeons’ prerequisites. Children naturally develop fine motor skills within the first years of life, regardless of surgical status. This may explain why longer follow-ups resulted in better functional outcomes despite dissatisfying primary surgical results with unstable, not satisfactorily growing phalangeal transfers. Disadvantages of an early age at operation include the risk of hypertrophic scars for any procedure and poor postoperative cooperation, which can impede recovery. Smaller anatomical structures lead to challenges in surgical technique. This is particularly important as children with symbrachydactyly may have hypoplastic, anomalous or absent nerves, blood vessels, and tendons. These structures must be of adequate size especially for vascularized transfers with microsurgical anastomosis, and failing revascularization has shown to be the main, early devastating postoperative complication in included studies [17, 20, 31, 68]. Apart from these statements, no definitive conclusion whether an early operation is beneficial was possible, due to inconsistent outcome reporting and variable age of primary operations.

Overall, limited evidence was available to conclude on general surgical strategies of symbrachydactyly treatment. Only retrospective cohort studies and case series were available with often insufficient or not adjusted outcome measurements for comparison with other series.

Only one study was identified comparing the surgical treatment options for children with symbrachydactyly. This is due to the fact that the disease is rare and most specialized surgeons and authors are in favour of one treatment method. Furthermore, the small number of patients prevented a direct comparison of post-operative outcomes between patients with different treatment options.

Among the included studies, patients with different diagnoses were included, and, thus, the individual impact of symbrachydactyly cannot be made. The patients were recruited over a period of 50 years and the different classifications over time, and regions render accurate reporting and classification of symbrachydactyly difficult. This is a realistic representation of how surgery evolves over time for rare and complex conditions, but this review summarizes the best available evidence to help guide clinicians.

There is considerable heterogeneity between studies and bias, which results in the low quality of included studies (scoring on average 38/100 points using the Modified Coleman Methodology Score). A distinction of case series to cohort studies was difficult, even if the suggestions by Mathes and Pieper [51] were followed. Studies comparing different surgical techniques or the implication of a worldwide database in order to directly compare outcomes would be valuable to determine which surgical procedure should be applied on which symbrachydactyly patients. Based on the findings of this review, the authors believe that there may be a justification to randomize patients in future studies.

## Conclusion

There is a lack of evidence for superiority of one surgical technique over another in the management of children with symbrachydactyly. Lengthening short fingers is the key challenge for functional improvement of a grasping hand. The investigated surgical techniques have individual strengths and weaknesses. Therefore, a tailored treatment approach to each patient, considering complications, the socioeconomic environment, capabilities of surgeons, and wishes of the parents, is the standard of care until future therapeutic alternatives are available [55].

### Supplementary Information

**Additional file 1: Supplement 1. **Literature search strings.** Supplement 2. **The Modified Coleman Methodology Score of the included studies.** Supplement 3.**Certainty of evidence assessment based of GRADE. **Supplement Table 1.**  Function, aesthetic and lengthening outcome measurements in *non-vascularized* transfers. **Supplement Table 2.** Function, aesthetic and lengthening outcome measurements in *vascularized *transfers. **Supplement Table 3.**  Function, aesthetic and lengthening outcome measurements in *distraction osteogenesis and web syndactyly release*. **Supplement Table 4.** Hand complications in *distraction osteogenesis* and *web release*. **Supplement 5.** Completed PRISMA Checklist.

## Data Availability

Not applicable.
