# Supplementary material for: Correction of symbrachydactyly: a systematic review of surgical options
Source: Syst Rev. 2023 Nov 16;12:218. doi: 10.1186/s13643-023-02362-7 (PMC10652478; doi:10.1186/s13643-023-02362-7)
Supplement: Supplementary file 1 — Additional file 1: Supplement 1. Literature search strings. Supplement 2. The Modified Coleman Methodology Score of the included studies. Supplement 3.Certainty of evidence assessment based of GRADE. Supplement Table 1. Function, aesthetic and lengthening outcome measurements in non-vascularized transfers. Supplement Table 2. Function, aesthetic and lengthening outcome measurements in vascularized transfers. Supplement Table 3. Function, aesthetic and lengthening outcome measurements in distraction osteogenesis and web syndactyly release. Supplement Table 4. Hand complications in distraction osteogenesis and web release. Supplement 5. Completed PRISMA Checklist. [file 13643_2023_2362_MOESM1_ESM.zip › Kopie von Symbrachydactyly_Supp_Tables.pdf]

Author (year)	Functional results hand	Aesthetic results hand	Aesthetic or functional results foot	Lengthening
Buck-Gramko (1990)	Mean ROM in new joint:<br>Age to 18 months: 35° (range 0°-90°)<br>19 months to 4 years: 10° (range 0° to 50°)<br>Over 4 years: 15° (range 0° to 70°).	Not reported.	Mobility of the donor toes was mostly limited in active flexion, but not in extension. Function feet was mostly not impaired.<br>Toe shortening:<br>- no shortening in 12 (17%)<br>- 3mm or less in 21 (30%)<br>- 4-7mm in 18 (26%)<br>- 8-12mm in 12 (17%)<br>- 9 toes in need of surgical treatment due to shortening (13%)	Age to 18 months: in 70% average 3mm growth. 30% no growth.<br>19 months to 4 years: Average 3,2mm growth. 15% radiologically open growth plates. 15% no growth.<br>Over 4 years: Average 1mm growth.
Cavallo (2003)	Mean ROM it the new joint was 60°.	Not reported.	Toe shortening:<br>- 3 mm or less in 46 (72%)<br>- 4-7mm in 15 (23%)<br>- >8mm in 3 (5%)<br>- Minor shortening in 93% middle phalangeal transfers.<br>- Minor shortening in 54% proximal phalangeal transfers.<br>Toe ROM:<br>- normal flexion in 60 patients (94%)<br>- active extension in 49 patients (77%).	Age to 18 months: Average resorption of 0.4mm<br>18 months to 4 years: 0.1mm resorption<br>Proximal phalanges transfer: average 0.7mm resorption<br>Middle phalangeal transfer: 0.8mm growth.
Garagnani (2012)	Not reported.	Not reported.	Emotional problems related to foot appearance were common. 100% tendency to hide feet. 7% hygiene issues. 7% cold intolerance, 3% balance problems. Instability of donor toes universal.<br>Oxford Ankle Foot Questionnaire:<br>- 89% parents dissatisfaction<br>- 93% patient dissatisfaction	Not reported.
Gohla (2005)	Instability (n= 6).<br>Range of motion was modest.<br>The improvement of hand function was remarkable in the most cases, yet not measurable.	Not reported.	Not reported.	Age to 18 months: 87% phalangeal growth, 4% resorption<br>18 months to 4 years: 49% phalangeal growth, 14% resorption<br>Over 4 years: 24% growth, 45% resorption
Kawabata (2018)	Active motion: good (n=24), fair (n=7), and poor (n=16).<br>Stability: good (n=37), fair (n=8), poor (n=2)<br>Alignment: good (n=33), fair (n=)5, poor (n=9).	Not reported.	Not reported.	Growth arrest at 5 years in 23%, and at 10 years, 78%. Gain in length of the transferred toe phalanx was 4.1 mm at 5-year follow-up and 5.2 mm at 10-year follow-up.
Leca (2008)	A) 1 phalanx resorbs other gets pinch MCPJ D2 15-0-19° ;<br>B) MCPJ D2 0-30-60°<br>C) 10-0-30° and 20-0-40° MCPJ movement after 2 FPT	Not reported.	Not reported.	1/6 non-vascularized phalangeal transfers resorption. No [illegible]
Sabapathy (2021)	Mean ROM at the MCP joint was -4° to 65° flexion. PIP joint complete stiffness (n=2), PIP-joint poor flexion (<30° flexion) (n=2).<br>Mean key pinch strength 1.3 kg (2.6 kg on the normal side). Children evaluated with Pediatric Outcomes Data Collection Instrument had high mean scores in all domains. The average PODCI scores were low for the upper-extremity domain. The mean PROMIS scores were within normal limits.	On the aesthetic component of the Michigan Hand Questionnaire, children gave higher scores than parents. Children gave a high score of 78.1/100, whereas parents gave a score of 63.3/100.	Donor toes were short in all children. No gait disturbance or toe instability. No difficulties in footwear, walking or running.	An open epiphysis was found in 24 of 31 grafts. The mean growth was 3.4mm. Length was 71.8% of the contralateral phalanx.
Unglaub (2006)	75% of the parents confirmed significant improvements of the functional performance and confirmed improvements in manual skillfulness of their child.	50% of parents confirmed improvements of self-confidence; 50% could not confirm improvements. 66% assessed the benefit of the operation and the postoperative procedure as justifiable.	15% minor problems. 10% putting on socks complicated. No functional disability.	Growth arrest:<br>Age to 18 months: 27%<br>18 months to 4 years: 10%<br>Over 4 years: 29%

Author (year)	Functional results hand	Aesthetic results hand	Aesthetic or functional results foot	Lengthening results
Foucher<br><br>(2001)	All patients <1 year of age had good integration, except for one “spur” with poor wrist mobility. Passive mobility in the 47 toes averaged 62° (range 32° to 72°). Active ROM mean of 38° and mean extension lag of 25°. The mean 2PD was 5 mm in 22 toes of 19 children over 7 years of age.	Not reported.	No morbidity in the donor feet. All were able to run, no neuromas. No cold intolerance.	4/65 premature growth plate closure. Growth follow up in 12 cases average similar to contralateral toe (-12% to +17%).
Hulsemann<br><br>(2002)	In 4/8 children without necrosis, a pincer grip with Maximal active span was in average 3,3cm. Pincer strength postoperative was in average 560g. In 6/8 children the 2-ponit-discrimination between 4-8mm.	Not reported.	No child had cosmetic issues concerning the foot modification. Normal footwear. One child had foot problems after several hour walks on asphalt.	Not reported.
Lister<br><br>(1988)	Active motion at the two interphalangeal joints has been achieved in 3 of 11 patients reviewed over the long term, totaling 35°, 40° and 40°, respectively. All showed sweating and good tactile adhesion by 6 months postoperatively.	Not reported.	The donor feet were of good appearance according to the surgeon. No difficulties.	Not reported.
Nikkhah (2016)	Satisfactory large grip function in all by ability to hold large play brick. 91% could perform fine, small and twist grip. No instability. Active ROM was generally poor. The passive ROM was average 54° more than the active ROM. Light-touch sensation in all present. Mean S2PD of 5mm (4–6mm). Eleven out of 12 parents strongly agreed that their<br><br>child’s toe-to-hand transfer had improved function.<br><br>One parent disagreed (Patient H), as they felt their child bypassed the transferred toe. Eleven out of 12 parents reported feeling very satisfied with the results of surgery and also reported a<br>willingness to recommend similar surgery to other families. Eleven strongly agreed that their toe transfer had improved hand function. Seven reported that their hands worked ‘normally’, four reported minor functional difficulties, and one child reported major functional problems. Cold<br>intolerance was assessed using the Campbell & Kay classification system; seven children had no symptoms, and four reported having had some symptoms but they were not causing problems. One child experienced troublesome symptoms of cold intolerance.	Out of<br><br>12 parents, four were neutral, three agreed that it had<br><br>improved the appearance of the child’s hand, and five<br><br>strongly agreed that appearance had been improved.	No significant long-term, donor-site problems. Mild toe clinodactyly was noted in five out of 12 patients in the review clinic, but this did not cause any functional or aesthetic concern; there were no problems with gait, balance, or neuroma formation, nor were there any problems with cold intolerance. Two children complained of minor scar hypersensitivity. No concerns of donor site appearance. Mild toe clinodactyly was noted in five out of 12 patients in the review clinic, but this did not cause any functional or aesthetic concern; there were no problems with gait, balance, or neuroma formation, nor were there any problems with cold intolerance. Two children complained of minor scar hypersensitivity. Ten out of 12 children reported never having any issues with donor-site appearance,<br>including that of the scar. Two children had sensitivity, particularly when they wore shoes, however, none of the children were self-conscious about the donor site	On radiographic analysis, the majority of physes in the transferred toes remained open and there was near normal longitudinal growth. The length of the transplanted digit varied in control digit from 83%–98% compared with<br><br>the unoperated donor site
Richardson<br><br>(2003)	2 tinger stiff. 77% parents happy with function of the hand.	85% parents happy with appearance of hand. On the aesthetic component of the Michigan Hand Questionnaire, children gave higher scores than parents. 3/13 parents reported that their child hid their hand occasionally.	100% parents happy with function and appearance of foot donor site.	Not reported.
Schenker (2007)	Average pinch strength ratio 50.9%. All<br>patients had normal tactile sensibility in the trans-<br>ferred digits except two who underwent toe trans-<br>fer as adolescents. The postoperative<br>motor function after toe transfer was more vari-<br>able and was poor in one patients with exception-<br>ally weak pinch strength and limited active range<br>of movement. Two patients who had a toe transferred onto the first ray with-<br>out a competent basal joint had a very weak pinch grip (<10 N). With one exception, all patients could perform precision grip tasts with their operated hand.	Not reported.	Not reported.	Not reported.
Van Holder<br><br>(1999)	Mean extension deficit of 20° and active compound flexion of the PIP and DIP joints of 80°. In hands without fingers in which the two toes were transferred to create opposing pinch, a useful pinch was obtained in all. Sensation was reported by the patients as “normal” and similar to adjacent digits in those toes where both digital nerves were repaired. Even when nerve repairs were not possible the patients reported protective sensation.	In all cases nail growth was normal and without deformity. The children’s assessment of the aesthetic appearance of the transfers was satisfactory to good. In no cases was a prosthesis worn or requested by the patient or the parents.	In most cases the scar on the foot was regarded as hypertrophic by the surgeon, yet no foot problems were reported by the patient.	In all cases growth of the transferred toes was comparable with growth of the remaining toes and the other transferred toes. No growth arrest was recorded. No correlation of growth with ischaemia or nerve repair was seen.

Author (year)	Functional results	Aesthetic results	Lengthening results
**Distraction osteogenesis**			
Foucher (2001)	Author comment that slender digits may experience stiffness.	might get stiff and slender digits, not necessarily good outcome	41 cases with 2.3cm length gain
Hierner (1998)	Lengthening does not provide normal circumference or interphalangeal joint motion.	Fibrosis of soft tissues: None (n=3), little (n=3), moderate (n=3)	Average finger extension 20.4mm.
Mann (2016)	Not reported.	Not reported.	With respect to preoperative length: Metacarpal average 18.4mm (9-40mm) =73%. Phalange distraction average 14.0mm (7-17mm) =77%. In 89% the distraction aim could be achieved.
Matsuno (2004)	No pinch at the follow-up evaluation. The range of motion was preserved to within 10° in all patients.	2 cases of symbrachydactyly, however, the bone growth was different not only for the lengthened metacarpals but also for the other metacarpals in the hand, thus causing the hand shape to change undesirably.	48% increase in lengthening with average 8.1mm distraction length.
Miyawaki (2002)	Pinch power in the affected hand was improved in all patients. The mean pinch power between the thumb and the elongated fingertip was 1.73 kg (range, 0.4 to 2.2 kg) on the involved side, compared with 1.70 kg (range, 0.3 to 2.2 kg) on the contralateral side. Careful examination demonstrated intact sensation throughout the lengthened rays.	Not reported.	No growth disturbance compared with the corresponding bones in the contralateral, normal hand at a mean of 3.9 years (range, 2-12 years) postoperatively. The bones were lengthened by a mean of 22.3 ± 4.2 mm (range, 20 to 28 mm), with a mean increase in bone length of 81.6% ± 21.5% (range, 50% to 115%).
**Web reconstruction**			
Deutinger (1989)	Full extend of flexion and extension was achieved in 20/22 hands. full extent of flexion in 13/19 hands, in 6 hands the range of flexion was incomplete	Not reported.	Not reported.
Iba (2012)	Tape measure test(Iba et al., 2011) 0.5 preoperative to 4.5 postoperative. Parents' assessment function markedly improved.	Not reported.	Not reported.
Li (2013)	Parents of 94.1% of the patients were satisfied with the overall function of the hand.	Parents of 76.5% were satisfied with the cosmetic appearance of the hand.	Not reported.
Shen (2022)	The mean (passive) width of the first webspace was 4.2 cm (range 3.5 to 5 cm).	Scarring assessed with the Vancouver Scar Scale showed a median value of 4 (range 0 to 7). Supple normal or hypopigmented scar (n=14). Mild hypertrophic scarring (n=2).	Not reported.

Author (year)	Complications hand
**Distraction osteogenesis**	
Foucher (2001)	31.7% complications: delayed unions, infection, dislocation, pain, too short, joint stiffness, clynodactyly, fracture
Hierner (1998)	44% complications: infection (n=1), early consolidation (n=1), excessive pain (n=1), refractures (n=3), axis rotations (n=2).
Mann (2016)	30% complications. Early consolidation (n=10), deviation (n=2), joint dislocation (n=1), pin infection (n=4), tendon dislocation (n=1), late consolidation (n=2) 16 reoperations were needed.
Matsuno (2004)	67% complications: Painful bony prominence of the fingertips (n=2).
Miyawaki (2002)	14% complications: Fracture of the fifth metacarpal (n=1).
**Web reconstruction**	
Deutinger (1989)	17% complications: Recurrence of syndactyly (n=5).
Iba (2012)	No complications reported. One patient required secondary additional first metacarpal rotation osteotomy to reconstruct an opposing position
Li (2013)	18% complications: scar tissue contracture due to partial necrosis of the local skin flaps (n=6).
Shen (2022)	No complications reported.
