# Supplementary figures and images for: Correction of symbrachydactyly: a systematic review of surgical options
Source: Syst Rev. 2023 Nov 16;12:218. doi: 10.1186/s13643-023-02362-7 (PMC10652478; doi:10.1186/s13643-023-02362-7)

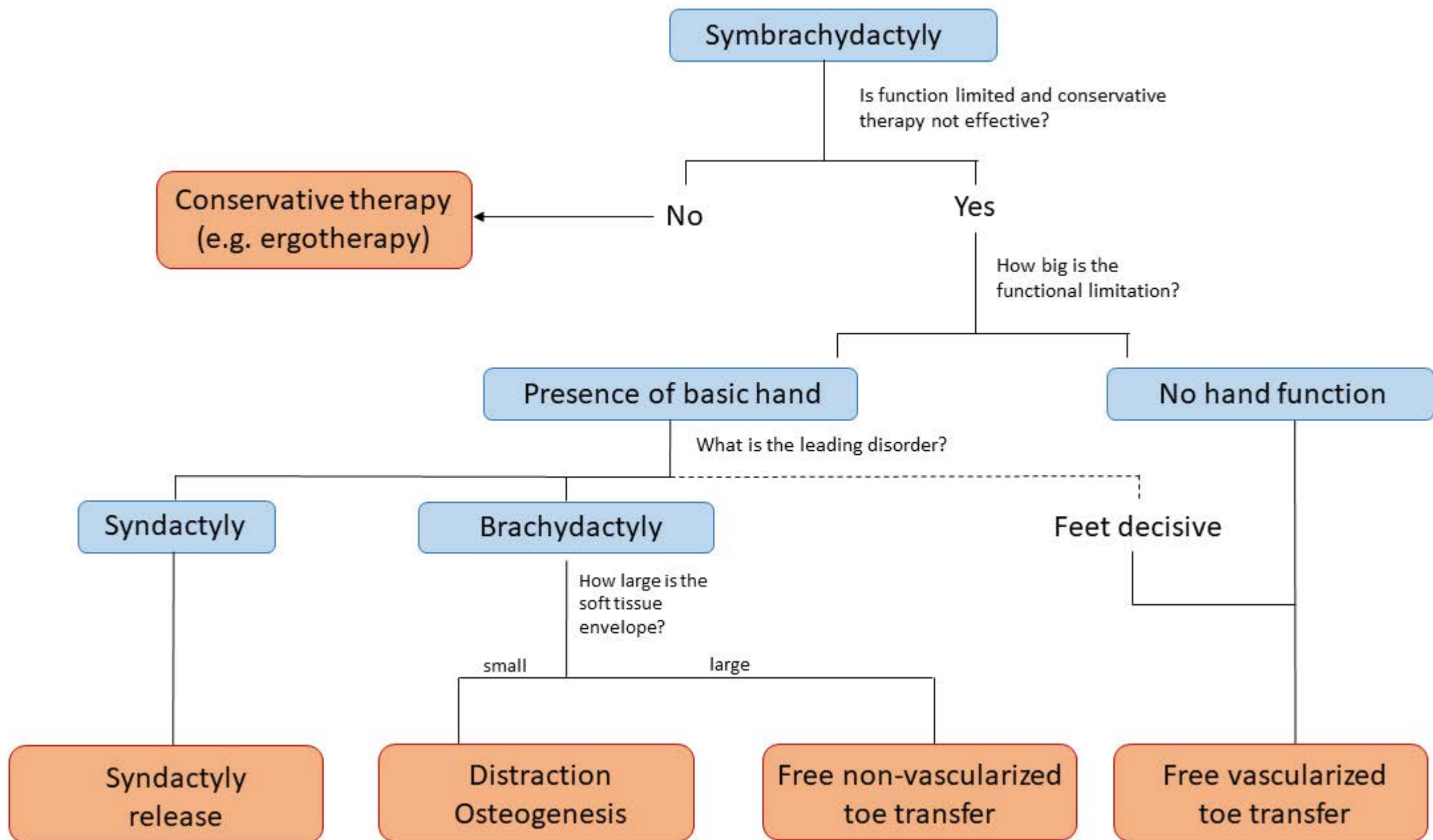

Supplement: Supplementary file 1 — Additional file 1: Supplement 1. Literature search strings. Supplement 2. The Modified Coleman Methodology Score of the included studies. Supplement 3.Certainty of evidence assessment based of GRADE. Supplement Table 1. Function, aesthetic and lengthening outcome measurements in non-vascularized transfers. Supplement Table 2. Function, aesthetic and lengthening outcome measurements in vascularized transfers. Supplement Table 3. Function, aesthetic and lengthening outcome measurements in distraction osteogenesis and web syndactyly release. Supplement Table 4. Hand complications in distraction osteogenesis and web release. Supplement 5. Completed PRISMA Checklist. [file 13643_2023_2362_MOESM1_ESM.zip › Symbrachydactyly treatment algorithm.pdf]

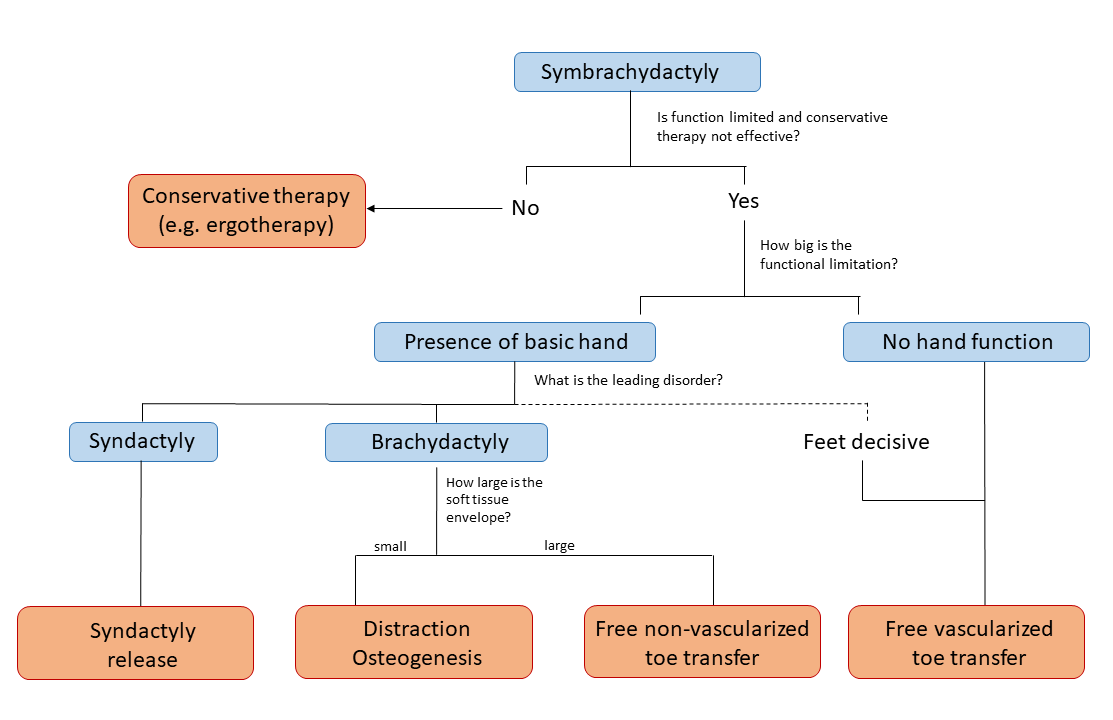

Supplement: Supplementary file 1 — Additional file 1: Supplement 1. Literature search strings. Supplement 2. The Modified Coleman Methodology Score of the included studies. Supplement 3.Certainty of evidence assessment based of GRADE. Supplement Table 1. Function, aesthetic and lengthening outcome measurements in non-vascularized transfers. Supplement Table 2. Function, aesthetic and lengthening outcome measurements in vascularized transfers. Supplement Table 3. Function, aesthetic and lengthening outcome measurements in distraction osteogenesis and web syndactyly release. Supplement Table 4. Hand complications in distraction osteogenesis and web release. Supplement 5. Completed PRISMA Checklist. [file 13643_2023_2362_MOESM1_ESM.zip › Symbrachydactyly treatment algorithm.png]
